# Are the Effects of DES Over? A Tragic Lesson from the Past

**DOI:** 10.3390/ijerph181910309

**Published:** 2021-09-30

**Authors:** Pilar Zamora-León

**Affiliations:** Department of Preclinical Sciences, Faculty of Medicine, Universidad Católica del Maule, Talca 3460000, Chile; pzamora@ucm.cl

**Keywords:** diethylstilbestrol, DES, endocrine-disrupting chemical, pregnancy, offspring outcomes

## Abstract

Diethylstilbestrol (DES), a transplacental endocrine-disrupting chemical, was prescribed to pregnant women for several decades. The number of women who took DES is hard to know precisely, but it has been estimated that over 10 million people have been exposed around the world. DES was classified in the year 2000 as carcinogenic to humans. The deleterious effects induced by DES are very extensive, such as abnormalities or cancers of the genital tract and breast, neurodevelopmental alterations, problems associated with socio-sexual behavior, and immune, pancreatic and cardiovascular disorders. Not only pregnant women but also their children and grandchildren have been affected. Epigenetic alterations have been detected, and intergenerational effects have been observed. More cohort follow-up studies are needed to establish if DES effects are transgenerational. Even though DES is not currently in use, its effects are still present, and families previously exposed and their later generations deserve the continuity of the research studies.

## 1. Introduction

**Figure 1:**
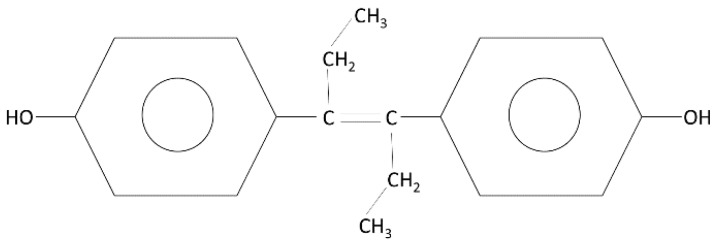
General structure of diethylstilbestrol (DES).

Diethylstilbestrol (DES; Figure 1) is an artificial hormone synthesized by Dodds and colleagues [1]. This synthetic nonsteroidal estrogen was synthesized by several different pharmaceutical laboratories around the world. DES was not patented due to public funding of the research. Dodds reported that DES could act as an agent to prevent pregnancy or induce abortion in rats and rabbits and, consequently, DES could be used as a contraceptive or emergency contraceptive pill. However, considering the possible adverse side effects in women, he censored the use of DES, suggesting that it might even induce cancer [2].

DES was first used for the treatment of estrogen deficiencies, such as vaginitis and menopausal symptoms, and for the suppression of postpartum lactation. In addition, it was prescribed to pregnant women from 1938 to 1971 to prevent miscarriages or premature deliveries in the US [3,4,5], even though Dieckman [6] presented studies in 1953 showing that DES did not reduce them. DES was prescribed for much longer in other European and American countries [7]. Lower-income countries constitute a major concern since DES was inexpensive, a factor that contributed to the use of this drug even after its withdrawal by the US Food and Drug Administration (FDA). This estrogenic endocrine-disrupting chemical (EDC) was forbidden because it caused clear-cell adenocarcinoma (CCA) of the vagina and cervix in young women (DES daughters) whose mothers took DES while pregnant (DES mothers). DES was used until 1978 to suppress postpartum lactation and until 1977 to treat menopause symptoms, as a postcoital contraceptive, and as a treatment for advanced postmenopausal breast cancer [3,8,9]. Since then, it has been replaced by tamoxifen, a drug with better results, to treat breast cancer, although in some cases, DES was used for this purpose for a longer time [10]. In addition, DES was used until 1985, when it was replaced by leuprolide to treat advanced prostate cancer. However, DES is still used in lower and middle-income countries as a treatment for castration-resistant prostate cancer [11].

The FDA banned DES because of its carcinogenicity in September 2000 [2,3,5]. It is classified as Group 1 human carcinogen by the International Agency for Research on Cancer (IARC) [4,5]. It has been estimated that orally administered DES is five times more potent than estradiol [12].

In addition to its use in humans, DES was utilized for growth in food-producing animals. However, because of risks to human health, it has been banned in several countries since 1979 [13,14,15,16,17]. Unfortunately, according to the study by Loizzo et al. [18], high levels of DES were detected in homogenized baby foods in Italy. The results suggested that DES was subcutaneously or intramuscularly implanted into animals, remaining in tissues for longer periods. This type of exposure of infants to DES could explain the increased detection of gynecomastia and precocious pseudopuberty in children. Additionally, traces of DES have been detected in livestock, meat, milk, fish, shrimp, river water, and sewage treatment plants [19,20,21,22,23,24,25].

Even though the metabolism of DES differs between animal species, it is mostly readily metabolized and efficiently cleared from the body. Urinary excretion is the predominant form of elimination in humans [26]. Even so, DES produces long-term pathophysiological effects. It has been found that early DES exposure is able to reprogram mouse reproductive organ development, affecting its responses to physiological estrogens [27,28,29,30].

The purpose of this report is to review the literature on the first nonsteroidal synthetic estrogen used in human hormone therapy [1] that led to one of the most tragic events in the history of reproductive medicine, as its effects are still present. First, the most common and relevant health effects induced by DES are summarized, focusing mainly on the female lower genital tract and breast cancer. Then, the effects induced in the third generation are investigated because not only pregnant women and their children, but also their grandchildren, have been affected. Currently, human studies in the third generation contain preliminary data. DES-induced epimutations have been reported, and the results indicate that the effects are due to intergenerational inheritance. Unfortunately, awareness of the DES tragedy has diminished in the population. Therefore, it is important to highlight and weigh the evidence since even though DES is not in use, its effects are not necessarily over yet. DES exposure is a good model to improve the current understanding of the effects of estrogens on hormonal imprinting, reproductive system development, and carcinogenesis. It is also a good model to evaluate the toxic effect that other pregnancy drugs that were, and are still, utilized can induce since they can be associated with different pathologies without a clear inheritance.

## 2. Relevant Health Effects Induced by DES

### 2.1. DES and Abnormalities in the Reproductive Tract

Several human studies have reported different adverse health outcomes induced by high early in utero DES exposure, such as structural anomalies of the cervix, uterus, and fallopian tubes, leading to difficult pregnancies, infertility, spontaneous abortion, preterm delivery, and stillbirth. However, some structural changes are harmless. Additionally, early menopause (before 45 years old) and CCA of the vagina and cervix have been described. The risk was higher for women with vaginal epithelial changes, a histological marker of exposure to high doses of DES [3,4,7,8,31,32,33].

DES sons present fewer genital abnormalities compared to DES daughters. The more common anomalies were hypospadias, hypoplasia of the penis, and undescended testicles (cryptorchidism) [3,33,34,35,36]. There is controversy with respect to DES exposure and infertility because some studies have found a lower sperm count and infertility, and others did not [35,37]. Studies performed in rats have shown that neonatal exposure to DES altered sperm morphology, motility, and fertility [38], and adult rats subcutaneously injected with DES showed a decrease in spermatogonia, Sertoli and Leydig cells, which can lead to a reduction in fertility [39]. Inconsistent findings related to testicular cancer have been reported [5,8,40]. A more recent study, a meta-analysis of men exposed prenatally to DES, showed a three-fold increase in the development of testicular germ cell tumors [41]. However, a study by Strohsnitter et al. [42] indicated that men exposed in utero to DES did not show an increased risk of developing overall or prostate cancer. Surprisingly, they found a reduction in the risk of urinary system cancers.

### 2.2. DES and Female Lower Genital Tract Cancer

The first discovered negative effect induced by DES was the increase in adenocarcinoma of the vagina in young women; all of them were DES daughters. These unusual tumors occurred in a cluster of 15–22-year-old women diagnosed, fortuitously, at the same hospital [43]. This discovery helped to forward the knowledge of the detrimental effects induced by DES. Several other studies have reported an increase in the development of CCA of the vagina and cervix in DES daughters [44,45,46,47]. In addition, the study by Troisi et al. [48] showed that DES daughters have an increased risk of developing a lower genital tract malignancy. In contrast, Palmer et al. [49] did not identify postnatal cofactors that could be associated with the risk of developing CAA in DES daughters.

The binding of DES to estrogen receptors (ER) also affects the expression of genes in the uterine myometrium, inducing its hyper-responsiveness to sex hormones, increasing the risk for uterine leiomyoma later in life and endometrial cancer in young and menopausal women [5,27,28,29,30]. Vaginal adenosis, a usual but non-obligatory precursor of CCA, is increased in women exposed to more elevated doses of DES [5]. Furthermore, Bromer et al. [50] found that in mice, after long in utero DES exposure, permanent hypermethylation of HOXA10 genes was induced, leading to abnormalities in the organogenesis of the uterus due to alterations during developmental programming.

In addition, DES daughters are at higher risk of presenting abnormal columnar epithelium of the cervix and upper vagina, developing vaginal adenosis or cervical intraepithelial neoplasia [32,51]. Furthermore, the study by Titus-Ernstoff et al. [52] reported an increase in ovarian cancer in DES daughters.

### 2.3. DES and Breast Cancer

DES is the best-characterized xenoestrogen associated with an increased risk of developing breast cancer in pregnant women and women exposed in utero. It has been determined that direct DES exposure induced a moderate increase in the risk of developing breast cancer, and the risk rises over time [47,53]. Also, Calle et al. [54] reported an increase in fatal breast cancers. On the other hand, a study by Colton et al. [55] showed an increase in the risk of developing breast cancer but found that the risk did not increase over time.

In the study by Palmer et al. [56], a cohort of women over 40-years-old exposed in utero to DES also presented an elevated risk of developing breast cancer. For the entire cohort, the risk was higher for estrogen-positive tumors. It was suggested that the effect of DES would increase as those women aged and approached the decades with higher breast cancer incidence [57]. Furthermore, the study by Tournaire et al. [58] also found a significant increase in breast cancer in women younger than 40 years. Additionally, a 10-year follow-up study of DES daughters showed an excess risk of breast cancer [48]. However, the study by Strohsnitter et al. [59] did not find any association between prenatal low-dose DES exposure and an increase in mammographic density, which is associated with a higher risk of developing breast cancer later in life. The authors did not discard the possibility of an association with higher doses of DES. A connection between higher doses of DES and breast cancer has been previously reported [32].

Research in rats has shown that DES exposure during pregnancy induced benign or malignant mammary lesions in the offspring [60,61]. Moreover, a study by Wormsbaecher et al. [62] demonstrated that in mice, in utero DES exposure induced stiffness and stromal alterations in the mammary gland in adult animals, which are considered risk factors for developing breast cancer in women. In addition, neonatal mice exposure resulted in more dilated ducts, terminal ducts hyperplasia, and a decrease in the number of mammary lobules [63]. Additionally, breast tumors have been described in the offspring of DES-exposed rats [4,60,64]. A relevant study on DES-induced alterations in mammary tissue was performed by Umekita et al. [65]. The group analyzed the gene expression profile of terminal end buds (TEBs) in rat mammary glands that were neonatally exposed to different doses of DES. The results suggested that changes in the expression of genes related with differentiation and development induced an increment in the number of TEBs during the period of higher vulnerability to the carcinogen, favoring uncontrolled and malignant cell proliferation.

Hilakivi-Clarke [66] suggested that DES exposure during pregnancy induced epigenetic alterations in genes associated with breast cancer during fetal development since the expression of several miRNAs decreases and the expression of DNA methyltransferases and histone deacetylases increases. The epigenetic silencing of miRNAs is probably part of the mechanism that increases 2-fold the risk of developing breast cancer in daughters. In addition, the study by Hsu et al. [67] specifically reported an epigenetic downregulation of miRNA-9-3 in breast epithelial cells exposed to DES, which leads to hypermethylation of its promoter, probably leading to the proliferation of breast cancer cells. It is important to determine if the altered epigenome is effectively increasing the vulnerability to breast cancer and, additionally, if those epimutations are reversible.

Other alterations in the epigenome have been described in MCF-7 cells in mice exposed in utero to DES, such as the increase of the Enhancer of Zeste Homolog 2 (EZH2), a histone methyltransferase that has been linked to breast cancer risk, and the increase in Histone H3 trimethylation [68]. In addition, the study by Bhan et al. [69] showed that the transcription of the long non-coding RNA HOTAIR, which is regulated by estradiol and plays a role in gene silencing and breast cancer, is affected by DES exposure in MCF-7 cells and rat mammary gland, by altering the epigenome of HOTAIR promoters.

### 2.4. DES and the Third Generation

The primary sources of information on the effects of DES in the third generation (F2; F0 = exposed females) come from murine studies, which indicate greater susceptibility to malignant tumor formation in the female reproductive tract and an increase in tumors as they age. Fertility was not affected, independent of DES exposure timing, even though it was reduced in F1 female mice [70,71]. In addition, the study by Walker [72] also showed uterine adenocarcinomas and ovarian cystadenocarcinomas in F2 female mice, and their development was associated with aging. Furthermore, he registered differences between F1 and F2 female mice with respect to the type of tumors and abnormalities. In the case of F2 male mice, similar results have been obtained, such as malignant tumor formation in the reproductive tract and proliferative lesions of the rete testis, but no apparent alterations in fertility were observed [73].

At this time, human studies showing the effects of DES in the third generation contain only preliminary data, as the grandchildren are reaching the age of cancer incidences. The potential effect induced by this EDC in the third generation has been considered in different reports because heritable alterations could be the result of epigenomic modifications induced by DES exposure during pregnancy [8,74,75,76].

Titus et al. [77] showed that DES granddaughters have irregular menstruation periods and amenorrhea, consistent with previous studies [31]. This is even more evident in DES granddaughters whose mothers, DES daughters, present alterations in the vaginal epithelium. This suggests that these changes may serve not just as a histological marker of high DES exposure but also as a marker of the effects of this EDC on the developing fetus. Moreover, these results imply epigenetic modifications of primordial germ cells of DES-exposed fetuses and the effects of DES as an endocrine disruptor. The results suggest an association with a higher percentage of infertility in DES granddaughters, but more research is needed. Fortunately, no significant increase in cancers of the female genital tract have been detected in DES granddaughters, but the cohort is still young, so follow-up is required [76]. Nonetheless, a case report study of an 8-year-old DES granddaughter with a history of dramatic vaginal bleeding and CCA of the vagina and cervix was described by Gaspari et al. [78], but the authors were unable to demonstrate a direct link between the grandmother’s exposure to DES and the development of this cancer. In addition, another case report of a 15-year-old DES granddaughter with small-cell carcinoma of the ovary was described, suggesting epigenomic modifications induced by DES exposure [79].

On the other hand, the study by Kaufman & Adam [30] did not find anomalies or neoplasms in the lower genital tract of DES granddaughters (between 15–28 years old), nor differences in the average age of menarche. One concern of this report is the low number of granddaughters, only 28. Another study showed that there are no differences in the age of menarche between granddaughters of prenatally DES-exposed and unexposed mothers [80]. Furthermore, Titus-Ernstoff et al. [52] found no overall increase in cancers in DES granddaughters, although ovarian cancer incidence was higher than expected.

For DES grandsons, investigations have shown an increase in hypospadias, even though the absolute risk is low [76,81,82]. In addition, hypospadias is more commonly found in DES grandsons born to DES daughters than in DES sons [83]. Probably, DES daughters presented hormonal alterations that lead to this anomaly during pregnancy due to epimutations induced by this EDC, among other reasons. In addition, the study by Tournaire et al. [36] showed an increase in cryptorchidism and hypoplasia of the penis. Unfortunately, the report did not have a correct control (sons of unexposed men). It is possible that cryptorchidism can predispose to testicular cancer [84]. In addition, they did not find genital alterations in daughters of DES sons, a relevant difference with the results obtained in female mice from prenatally exposed males [85]. Furthermore, the study by Gaspari et al. [86] suggested a relationship between DES prenatal exposure and the development of “idiopathic” partial androgen insensitivity syndrome, a genetic condition that leads to low response to male sex hormones in DES grandsons.

Furthermore, Tournaire et al. [76] reported an increase in orofacial clefts, esophageal abnormalities, musculoskeletal and heart defects, and cerebral palsy associated with increased premature births in DES grandchildren. Moreover, the observational study by Kioumourtzoglou et al. [87] found an increase in the risk of developing attention-deficit/hyperactivity disorder (ADHD) in DES grandchildren if DES was taken in the first trimester of pregnancy. It is possible that other factors could be associated with the increased risk.

In general, human studies usually consider a low number of participants, complicating the understanding of the outcomes.

## 3. Discussion

It has been known for decades that xenoestrogens are associated with the development of tumors. Since the late 1930s, studies in mice have shown that DES exposure could induce neoplasms in genital and breast tissues. Unfortunately, the experimental results were ignored, and the FDA approved the use of DES as a “safety drug”. DES has the ability to cross the placenta, altering the development of the fetus, behaving as an endocrine disruptor, teratogen, and carcinogen [3,4,50]. DES can induce severe alterations in the reproductive tract of the fetus that can lead to disease decades later. Unfortunately, after so many years, identifying the detrimental effects is challenging.

The general risk of cancer in DES mothers is low, but several reproductive and structural problems have been described in DES daughters and DES sons [8,32,33,41,42,73,77,81,88]. In addition, there appears to be an association between timing and dose of in utero exposure and the risk of more detrimental outcomes during puberty [89,90].

Mice and rats are good animal models for studying prenatal DES exposure because of their similarities to humans [38,63,64,65]. Results have demonstrated that in utero DES exposure leads to infertility, anomalies in the reproductive tract and breast, and non-neoplastic and neoplastic tumor development, mimicking the effects induced by DES in humans. In addition, the effects have been evaluated in multiple rodent generations, helping to predict the effects in grandchildren. For instance, the reduced fertility detected in DES F1 female mice was not observed in the F2 generation, but an increased vulnerability to neoplasia was transmitted [71], correctly predicting the outcomes in humans. In addition, the menstrual irregularities observed in granddaughters could imply a higher risk of developing ovarian cancer or being infertile.

Studies have been performed to understand the modifications in signaling pathways induced by prenatal DES exposure, but the molecular mechanisms that lead to an increased risk of cancer and other physiological alterations remain unclear [45,65]. The effects induced by DES are important to understand because many xenoestrogens polluting the environment have similar effects. Common molecular pathways are altered, leading to breast and reproductive tract cancers and other health problems [91,92]. DES, like other EDCs, can enter our bodies not only as a drug but also as a toxic pollutant and through the food chain. In this manner, humans can be dangerously affected. DES was withdrawn from the market as a growth stimulator for livestock and other animals, but it is difficult to know how rigorous the controls are around the world. Moreover, the subsequent development and use of other growth stimulator compounds for farm animals may affect molecular signaling pathways similarly to DES. Additionally, DES and other EDCs, by affecting the appropriate development of the reproductive tissues, may alter the response to endogenous estrogens during puberty or adulthood. Even though the environmental concentrations of the EDCs are not as high as DES doses, the combination of all of them could be synergistic.

It has been estimated that over 10 million people worldwide have been exposed in utero to DES [7,9]. Although DES has not been prescribed for about 50 years, its adverse effects appear to still be present in the third generation. Several studies have suggested that epigenetics plays a role through modifications in the germline, which means that the number of possible affected individuals is high [78,93,94,95,96].

As previously mentioned, DES granddaughters have irregular menstruation periods and amenorrhea; a case of CCA of vagina and cervix of an 8-year-old girl, and small-cell carcinoma of the ovary of a 15-year-old girl have been described [31,77,78,79]. However, although CCA is a rare gynecological cancer, other cases have been reported in young women with no history of DES exposure [97]. The question that arises is whether they were exposed to other EDCs that could induce the activation of molecular pathways similar to DES. In the case of DES grandsons, increases in hypospadias, cryptorchidism, and hypoplasia of the penis have been reported [36,76,81,82,83]; also, “idiopathic” partial androgen insensitivity syndrome has been described [86]. The alterations in the genital tract of DES grandsons could be associated with hormonal imbalances in DES daughters, placental malfunctions affecting normal fetal development, and modifications in genes involved in the development of the genital tract, such as HOXA and ER-α genes [81,82]. However, most studies have been performed on DES daughters and only a few concern DES sons. More studies are needed that focus on the inheritance of DES sons. Additionally, besides genital tract and breast anomalies, other physiological alterations in grandchildren have been reported, such as neurodevelopmental and socio-sexual behavior effects, as well as immune, pancreatic and cardiovascular disorders [3,76,87,88,98,99,100]. All of these findings suggest inherited epimutations due to in utero DES exposure.

Exposure to DES during pregnancy affected three generations simultaneously: the mother (F0), the fetus (F1), and its germ cells (F2). A summary of DES-induced clinical effects in humans is described in Table 1.

**Figure 2:**
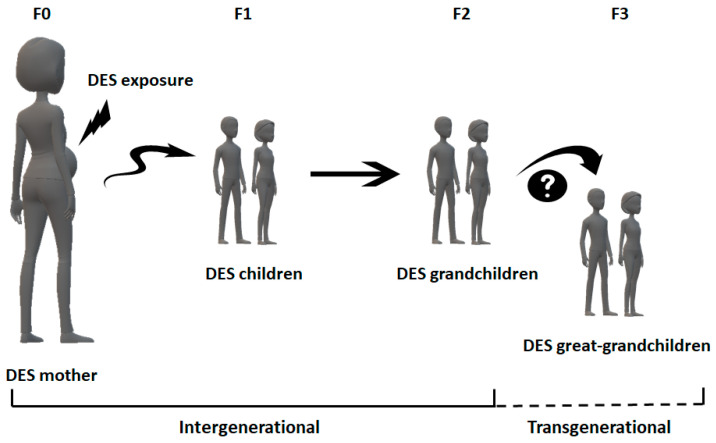
Intergenerational and transgenerational DES effects. The solid line represents what is known, and the dashed line, what is still hypothetical.

This indicates that the changes in the epigenome are due to intergenerational inheritance and not to transgenerational inheritance, as mentioned in several reports. Effects on the fourth generation (F3) are required to classify the effects of DES as an epigenetic transgenerational inheritance (Figure 2). Additionally, primary epimutations in germ cells should induce a characteristic phenotype in the progeny. Great-grandchildren are the only ones not directly exposed to this EDC or through germ cells [94,95,96,101,102,103]. It is too soon to know if DES effects are transgenerational since great-grandchildren are still too young (DES-exposed women are approximately 60 years old) or, at least, no reports have been yet published.

It is worth depending on animal research as bona fide approximations. Mice are good for inter-and transgenerational studies because at least three generations can be observed and evaluated during a year. Moreover, if DES multigenerational neoplastic effects observed in rodents also occur in humans, it will take about five decades to identify the effects in future generations [104]. If this is the case, the story of DES is not over yet.

The “mark” that DES could have left in the epigenome should be associated with the time of the exposure and the doses. The vulnerable in utero periods are relevant for the possible development of disease in the offspring later in life because the response to xenoestrogen could or could not be irreversible. Additionally, if the placenta is affected by DES exposure, the impacts on fetal development would be different depending on the sex of the fetus. Once the epimutations induced by the EDC become “stable” (escape epigenetic reprogramming), epigenetic transgenerational inheritance is established, increasing disease vulnerability in the next generations.

In addition, it is important to consider that individuals may be exposed to toxic environmental pollutants, such as bisphenol A or others later in life. These additional exposures may affect the risk of disease due to secondary epimutations, complicating the understanding of the outcomes of DES exposure in future generations [94,96,98,101,102,103,105]. Therefore, developmental features of disease etiology and their association with epigenetic transgenerational inheritance are currently not clear. It is important to analyze the disease, inherited exposure, and epigenetics and find biomarkers to use as tools to diagnose disease vulnerability.

Research must continue in order to determine the generational impacts of DES. Follow-up cohort studies are required, and more DES sons and their children should be included. Different cohort studies now exist, and currently, the study by Hoover et al. [32] has been expanded to the third generation cohort. The National Cancer Institute (NIH) stated that its latest follow-up study began in 2016. In addition, the NIH has plans for a broader study of the effects of DES on genetic markers and hormone metabolism and began enrolling women in 2020 (https://www.desfollowupstudy.org/index.asp) (accessed on 5 July 2021). It is known that access to databases can be complicated, affecting the medical history of DES exposure. Unfortunately, awareness of the DES tragedy has declined in the population. Nevertheless, all efforts must be made to increase the robustness of the results and the understanding of this public health disaster.

It is important to evaluate pregnancy drugs that were used in the past as well as those currently being utilized since they can be associated with different pathologies with no clear inheritance, probably induced by germline toxicity. Transplacental exposure has to be evaluated, and the many possible risks should be considered. Research should then continue to establish the mechanisms of action of DES, or any other EDC, on specific tissue targets and germline epigenome for the development of future appropriate pharmaceutical pills. The molecular pathways associated with environmental toxic compounds and the modifications induced in the endocrine system leading to disease are important to identify. In addition, as research continues, we have to search for more possible extensive effects, not only the ones associated with the genital tract or breast [93,98].

## 4. Conclusions

In view of the tragic experience with DES, pregnant women should never be exposed to EDC unless strictly necessary. In addition, the absence of immediate toxicity is not enough to prove the possible beneficial properties of a drug because the effects could be observed in the next generations through epigenomic modifications. This lesson should be learned in order to prevent other future medical catastrophes. Moreover, research publications about the effectiveness or non-effectiveness of a drug should be highly considered. At least the strictness in the regulation and approval of new drugs has been improved, so this type of error is less probable to occur again. If DES had been withdrawn for pregnancy “treatments” when the first controversial experimental results were obtained, the tragedy could have been avoided for millions of mothers, their children, grandchildren, and maybe, great-grandchildren.

Considering all the aforementioned, it is important that gynecologists and pediatricians control and follow-up DES grandchildren and DES great-grandchildren. They should be informed and educated about the potential risks of developing cancer and other adverse health outcomes, and in that manner, prevent or reduce risks. Family medical history should be systematically considered, and information on hormone use and miscarriages would be helpful, especially when patients do not know if any of their ancestors were exposed to DES [33,90]. Even though DES is not currently in use, its effects are still present, and because of that, research fundings should continue. Families previously exposed and their later generations deserve it.

## Figures and Tables

**Table 1:** Human clinical effects induced by DES.

	Generation Exposed To Des	Human Clinical Effects	Authors
**Female abnormalities in the reproductive tract**	DES daughters (F1) and DES granddaughters (germ cells; F2)	28 DES granddaughters did not show abnormalities in the lower genital tract contrary to their DES mothers (high frequency).	[30] Kaufman et al.
	DES granddaughters (germ cells; F2)	DES granddaughters with irregular menstrual periods and amenorrhea; no risk of reproductive dysfunction.	[31] Titus-Ernstoff et al.
	DES granddaughters (germ cells; F2)	Increased risk of irregular menstrual periods (more common in DES granddaughters of DES mothers with vaginal epithelial changes) and amenorrhea. Possible increased risk of ectopic pregnancy.	[77] Titus et al.
	DES granddaughters (F2)	No differences in age at menarche between DES granddaughters and DES daughters.	[80] Wilcox et al.
**Female lower genital tract and breast cancers**	DES mothers (F0)	Modest increased risk of breast cancer (not aggravated by a family history of breast cancer, oral contraceptives, or hormone replacement therapy). No evidence of a risk for ovarian, endometrial, or other cancers.	[47] Titus-Ernstoff et al.
	DES mothers (F0)	Moderate increase in the risk of breast cancer (risk rises over time).	[53] Greenberg et al.
	DES mothers (F0)	Increase in fatal breast cancers (did not increase over time).	[54] Calle et al.
	DES mothers (F0)	Modest increased risk of breast cancer (statistically significant); it did not increase over time.	[55] Colton et al.
	DES daughters (F1)	High risk of cervical intraepithelial neoplasia and breast cancer (at 40 years or older). Also, early menopause, infertility, abortion, premature delivery, ectopic pregnancy, stillbirth. The risk was higher in women with vaginal epithelial changes.	[32] Hoover et al.
	DES daughters (F1)	Increase in adenocarcinoma of the vagina in young women (cluster of 15–22-year-old women).	[43] Herbst et al.
	DES daughters (F1)	Increased risk of CCA of the vagina and cervix, and breast cancer.	[44] Troisi et al.
	DES daughters (F1)	Increased risk of CCA of the vagina and cervix; marginally increased risk of melanoma (before age 40). No increased risk of breast cancer (cohort relatively young).	[45] Verloop et al.
	DES daughters (F1)	Excess risk of breast cancer, increased risk of lower genital tract malignancies (relatively small absolute risk), and pancreatic cancer. No increased risk of overall cancer.	[48] Troisi et al.
	DES daughters (F1)	No postnatal cofactors were identified in association with the risk of developing CAA.	[49] Palmer et al.
	DES daughters (F1)	Elevated risk of breast cancer only in women 40 years of age or older.	[56] Palmer et al.
	DES daughters (F1)	Increased risk of breast cancer after 40 years old.	[57] Palmer et al.
	DES daughters (F1)	Significant increase of breast cancer in women (younger than 40 years) and CCA of the cervix or vagina. No significant increase in overall cancer.	[58] Tournaire et al.
	DES daughters (F1)	No association between prenatal exposure to low doses of DES and increased mammographic density in premenopausal or postmenopausal women (did not discard the possibility of an association with higher doses of DES exposure).	[59] Strohsnitter et al.
	DES granddaughters (and DES grandsons) (germ cells; F2)	Moderate increase in the risk of breast cancer (risk rises over time). Increased risk of CCA of the vagina and cervix, and higher than expected incidence of ovarian cancer (3 cases). No overall increase of cancer risk in DES grandchildren.	[52] Titus-Ernstoff et al.
	DES granddaughter (germ cells; (F2)	Case report of CCA of the vagina and cervix of an 8-year-old girl (with a history of severe vaginal bleeding). DES mother had a hysterectomy.	[78] Gaspari et al.
	DES granddaughter (germ cells; F2)	Case report of a 15-year-old girl with small-cell carcinoma of the ovary.	[79] Blatt et al.
**Male abnormalities and cancers of the reproductive tract**	DES sons (F1)	DES sons showed an increased risk of urogenital abnormalities (strongest association with early gestational exposure).	[35] Palmer et al.
	DES sons (F1)	No increased risk of overall cancer in DES sons; testicular cancer may be increased in DES sons.	[40] Strohsnitter et al.
	DES sons (F1)	Threefold increase in testicular cancer.	[41] Hom et al.
	DES sons (F1)	Increased risk of hypospadias (few cases).	[82] Klip et al.
	DES sons (F1)	No increase in overall or prostate cancer. Unexpected reduction in the risk of cancers of the urinary system.	[42] Strohsnitter et al.
	DES grandsons (and DES granddaughters) (germ cells; F2)	Increased incidence of cryptorchidism and hypoplasia of the penis; no increased incidence of hypospadias. No increase of genital anomalies in girls. All grandchildren were born to DES sons.	[36] Tournaire et al.
	DES grandsons (germ cells; F2)	Increase in hypospadias in DES grandsons (born to DES daughters), even though the absolute risk is low; no mutations and no polymorphisms of the AR and MAMLD1 genes were found. Results based on few cases.	[81] Kalfa et al.
	DES grandsons (germ cells; F2)	Increased risk of hypospadias when DES grandsons were born to DES daughters but not to DES sons.	[83] Brouwers et al.
	DES grandsons (germ cells; F2)	11 DES grandsons with “idiopathic partial androgen insensitivity-like syndrome”.	[86] Gaspari et al.
**Other alterations**	DES daughters (F1)	Associations with coronary artery disease and myocardial infarction, but not with stroke.	[99] Troisi et al.
	DES children (F1)	Increased risk of pancreatic cancer in DES daughters but not in DES sons.	[100] Troisi et al.
	DES grandchildren (germ cells; F2)	Increased cerebral palsy, increased defects in lip/palate, esophagus, musculoskeletal and circulatory systems. Also, increased male genital tract anomalies. No significant abnormalities in female genital tract and no increase of breast, uterus, and ovary cancers.	[76] Tournaire et al.
	DES grandchildren (germ cells; F2)	Increased ADHD risk (only first trimester of DES exposure during pregnancy).	[87] Kioumourtzoglou et al.
	DES grandchildren (germ cells; F2)	Overall birth defects were elevated in grandchildren. Granddaughters appeared to have an increased risk of heart defects.	[88] Titus-Ernstoff et al.
